# Examining the Clinical Utility of Selected Memory-Based Embedded Performance Validity Tests in Neuropsychological Assessment of Patients with Multiple Sclerosis

**DOI:** 10.3390/neurolint13040047

**Published:** 2021-09-23

**Authors:** John W. Lace, Zachary C. Merz, Rachel Galioto

**Affiliations:** 1 Neurological Institute, Section of Neuropsychology, Cleveland Clinic Foundation, Cleveland, OH 44195, USA; galiotr@ccf.org; 2 LeBauer Department of Neurology, The Moses H. Cone Memorial Hospital, Greensboro, NC 27401, USA; zachary.merz@conehealth.com; 3 Mellen Center for Multiple Sclerosis, Cleveland Clinic Foundation, Cleveland, OH 44195, USA

**Keywords:** multiple sclerosis, neuropsychological assessment, noncredible performance, embedded performance validity tests

## Abstract

Within the neuropsychological assessment, clinicians are responsible for ensuring the validity of obtained cognitive data. As such, increased attention is being paid to performance validity in patients with multiple sclerosis (pwMS). Experts have proposed batteries of neuropsychological tests for use in this population, though none contain recommendations for standalone performance validity tests (PVTs). The California Verbal Learning Test, Second Edition (CVLT-II) and Brief Visuospatial Memory Test, Revised (BVMT-R)—both of which are included in the aforementioned recommended neuropsychological batteries—include previously validated embedded PVTs (which offer some advantages, including expedience and reduced costs), with no prior work exploring their utility in pwMS. The purpose of the present study was to determine the potential clinical utility of embedded PVTs to detect the signal of non-credibility as operationally defined by below criterion standalone PVT performance. One hundred thirty-three (133) patients (*M* age = 48.28; 76.7% women; 85.0% White) with MS were referred for neuropsychological assessment at a large, Midwestern academic medical center. Patients were placed into “credible” (*n* = 100) or “noncredible” (*n* = 33) groups based on a standalone PVT criterion. Classification statistics for four CVLT-II and BVMT-R PVTs of interest in isolation were poor (AUCs = 0.58–0.62). Several arithmetic and logistic regression-derived multivariate formulas were calculated, all of which similarly demonstrated poor discriminability (AUCs = 0.61–0.64). Although embedded PVTs may arguably maximize efficiency and minimize test burden in pwMS, common ones in the CVLT-II and BVMT-R may not be psychometrically appropriate, sufficiently sensitive, nor substitutable for standalone PVTs in this population. Clinical neuropsychologists who evaluate such patients are encouraged to include standalone PVTs in their assessment batteries to ensure that clinical care conclusions drawn from neuropsychological data are valid.

## 1. Introduction

Multiple sclerosis (MS) is a chronic, inflammatory, demyelinating condition that can present with diverse patterns of physical, cognitive, and psychiatric symptoms [1,2,3,4]. In addition to expert care provided by neurologists who may provide clinical monitoring and pharmacological intervention (e.g., dimethyl fumarate, interferons, monoclonal antibodies) [5,6], clinical neuropsychologists are often called upon to assess such patients and provide targeted treatment recommendations for them from a neurocognitive and psychological perspective [7,8]. Several attempts to standardize the clinical neuropsychological assessment of patients with MS (pwMS) have been made. The National MS Society provided practicable recommendations for cognitive screening in patients with MS [1]. Over the previous two decades, the Minimal Assessment of Cognitive Function in MS (MACFIMS) [9], the Brief International Cognitive Assessment for MS (BICAMS) [10], and the Mercy Evaluation of Multiple Sclerosis (MEMS) [11] have been proposed as compendious neuropsychological batteries for more thorough evaluation (see [11] for a review of similarities and differences among these batteries).

Clinical neuropsychologists are tasked with identifying bona fide neuropsychological dysfunction while ensuring that extraneous factors –which may negatively influence results and clinical conclusions—are considered. Notably, noncredible presentations of cognitive impairment can seriously reduce overall neuropsychological performance above and beyond disease- or injury-related variables [12,13]. Several case studies of noncredible cognitive presentations in pwMS have been reported and debated [14,15,16,17]. At a broader level, more than one-fifth of clinically-referred patients with MS have been reported to demonstrate noncredible performance [18,19], consistent with base rates in other clinically referred neuropsychological samples [20]. Despite there being “no generally accepted explanation for suboptimal cognitive performance in MS” [19] (p. 1), many have identified the presence of external incentive (e.g., receipt of disability insurance), significant psychological/psychiatric symptoms, and pain/fatigue as being potential contributing factors in this and other populations [21,22,23] while suggesting that the degree of the radiological disease may be relatively noncontributory to PVT performance in pwMS [18].

Importantly, clinical neuropsychologists must determine the validity of cognitive data—typically via standalone and/or embedded performance validity tests (PVTs)—to ensure clinical decisions and aspects of continued medical care for pwMS are pursued ethically and responsibly [24,25]. As an aside, experts have encouraged neurologists to become familiar with validity assessment methods to aid in the interpretation of neuropsychological test results [26]. Despite the reported base rates of noncredible performance in MS, none of the aforementioned neuropsychological batteries (nor the National MS Society recommendations) explicitly suggest the inclusion of a standalone PVT in clinical assessment. Only the paper describing the MEMS [11] discussed in clear detail the necessity of PVT use in this context, though it did not specifically suggest a standalone PVT in its battery. Thus, clinicians who utilize these tools are left with the task of evaluating the only available embedded PVTs to determine credibility.

In contrast to standalone PVTs, which require their own administration time and materials and tend to have improved psychometric properties, embedded PVTs offer their own balance of benefits and detriments [27]. On one hand, they tend to offer lower classification accuracies and sensitivities compared to their standalone analogs, with many embedded PVTs approaching the so-called “Larrabee limit” [28] of approximately 0.50 sensitivity when maintaining ideal specificity (i.e., ≥0.90). On the other hand, they may increase efficiency in neuropsychological testing procedures by reducing the duration of examination time and decreasing the overall test burden on examinees. This consideration is of clinical relevance given the frequency of reported fatigue in MS [1,10]. Each of the aforementioned batteries recommends the use of the California Verbal Learning Test, Second Edition (CVLT-II) [29] and Brief Visuospatial Memory Test, Revised (BVMT-R) [30], both of which are well-validated list-learning and visual learning/memory tasks (respectively) that include embedded PVTs [31,32]. Specifically, the CVLT-II includes embedded PVTs of Recognition Hits (C-RH) and Forced Choice Recognition (C-FCR), and the BVMT-R includes Recognition Hits (B-RH) and Recognition Discrimination (B-RD). Each of these variables has been supported as embedded PVTs in recent literature, mostly in individuals with traumatic brain injuries or mixed clinical samples [33,34,35,36,37].

Unfortunately, these embedded PVTs remain infrequently examined in the context of MS despite their potential for clinical value. To the authors’ knowledge, only Domen and colleagues [38] have explored this question, and they reported specificity values ≥0.92 for previously validated cutoffs for C-FCR and B-RD, suggesting that traditional cutoffs may not yield high rates of false-positive errors. Importantly, their findings are limited by their decision to exclude patients performing below expected limits on a standalone PVT and subsequently not reporting classification statistics besides specificity (i.e., sensitivity, classification accuracy).

As such, the present study examined the clinical utility of embedded PVT variables in CVLT-II and BVMT-R—recommended by many experts for routine use in pwMS—in identifying noncredible performance, operationally defined as below criterion performance on a well-validated, standalone, memory-based PVT, in a sample of pwMS. We evaluated select variables both in isolation and in combination (using both arithmetically- and logistic regression-derived formulas) to determine their possible utility.

## 2. Materials and Methods

### 2.1. Patient Characteristics

Patients with MS (pwMS) were identified by retrospective analysis of an archival clinical dataset of individuals referred for clinical neuropsychological assessment within a large, Midwestern, academic medical center. All patients underwent neuropsychological assessment between 2017 and 2021. Inclusion criteria were: (1) complete data for all variables of interest; (2) estimated premorbid intellectual ability, as measured by a word reading test, ≥70; and (3) most recent or only neuropsychological evaluation.

The sample included 133 patients (*M* age = 48.28, *SD* = 12.55; *M* Education = 13.96 years, *SD* = 2.49). Most patients were women (76.7%) and identified as White/Caucasian (85.0%). Most patients were diagnosed with a relapsing-remitting subtype (75.2%), with relatively fewer primary (15.0%) and secondary (9.8%) progressive subtypes. Patients (*n* = 118 with data for this variable) had an average disease duration (since reported symptom onset) of 13.34 years (SD = 10.53).

### 2.2. Measures

#### 2.2.1. Wide Range Achievement Test, Fourth Edition

The Word Reading subtest of the Wide Range Achievement Test, Fourth Edition (WRAT-4) [39] was used as an estimate of premorbid intellectual ability.

#### 2.2.2. Victoria Symptom Validity Test

The Victoria Symptom Validity Test (VSVT) [40] is a widely used, standalone, memory-based PVT. A recent systematic review and meta-analysis identified the best performing variable as the total score of ≤40 to indicate noncredible performance [41]. The VSVT was chosen for use in this sample of patients with MS because it is minimally affected by bona fide deficits in working memory, processing speed, and memory [42] and is not likely related to MS disease burden [18].

#### 2.2.3. Embedded PVTs

Forced Choice Recognition (C-FCR) and Recognition Hits (C-RH) from the CVLT-II [29] and Recognition Hits (B-RH) and Recognition Discrimination (B-RD) from the BVMT-R [30] were chosen due to their availability within the MACFIMS, BICAMS, and MEMS [9,10,11].

### 2.3. Procedures

Approval from the first and third authors’ institution’s IRB was obtained prior to data analysis. All patients were referred for neuropsychological assessment as part of medical care and were evaluated by a board-certified clinical neuropsychologist or clinical psychologist trained under Houston Conference Guidelines. All measures were administered by either a board-certified or trained psychometrist, clinical psychology doctoral student, or postdoctoral neuropsychology fellow proficient in test administration and scoring. Patients with VSVT Total Accuracy scores ≥41 were categorized as “credible” and those with VSVT Total Accuracy scores ≤40 were categorized as “noncredible” [41].

### 2.4. Statistical Analyses

All analyses were conducted with SPSS 26.0. Demographic variables were compared between credible and noncredible groups using *χ*^2^ or Mann-Whitney U tests. Embedded PVTs in isolation and arithmetically and logistic regression-derived formulas were evaluated. Five arithmetic formulas were computed to determine if combining variables resulted in improved classification. These combinations of variables were:

1. C-FCR + C-HR + B-HR + B-RD;
2. C-FCR + B-RH;
3. C-FCR + B-RD;
4. C-RH + B-RH; and
5. C-RH + B-RD.

Five logistic regressions (LRs) were performed with the combinations of variables described in the formulas above entered as predictors as one set (i.e., all four were entered as predictors for the first, C-FCR and B-RH were entered as predictors for the second, etc.) with credible/noncredible group membership as the binary outcome variable

Mann-Whitney U tests, areas under the receiver operating characteristic curve (AUCs), sensitivity, and specificity were calculated for PVTs in isolation using credible vs. noncredible group membership as the criterion variable. AUCs were additionally computed for each arithmetically- and logistic regression-derived formula. A conservative adjusted criterion α of 0.013 (i.e., 0.05/4) was used for *χ*^2^, Mann-Whitney U, and AUC analyses due to the abundance of repeated analyses to minimize the likelihood of Type 1 error. AUC values ≥0.70 suggest at least acceptable discriminability, and values below 0.70 indicate poor classification ability and are generally considered unacceptable [43].

## 3. Results

On average, the premorbid intellectual ability was in the average range (*M* WRAT-4 Reading Standard Score = 96.79, *SD* = 10.79). Regarding the VSVT, 100 (75.2%) patients were classified as having “credible” neuropsychological test performance and 33 (24.8%) were classified as having “noncredible” neuropsychological test performance [41].

Demographic characteristics for both groups are displayed in Table 1. The credible and noncredible groups did not significantly differ in terms of age, racial/ethnic identity (coded and White vs. Non-White), MS subtype, nor symptom duration (all *p*s ≥ 0.08). The credible and noncredible groups differed in terms of gender and years of education (*p*s ≤ 0.01). However, the magnitude (i.e., effect size) of the gender difference was negligible-to-weak (Cramer’s *V* = 0.23) [44], consistent with similar previous research [45], and was not considered to be impactful. Additionally, the noted difference in years of education between groups was not considered to be clinically meaningful, as has been the case with prior literature with similar aims [46,47].

Mann-Whitney U tests revealed that each of the four embedded PVT variables (C-FCR, C-HR, B-HR, and B-RD) were not significantly different between groups (all *p*s > 0.017), with notably small effect sizes (*d*s ranged from 0.22–0.37). AUCs for each of the four embedded PVT variables ranged from 0.58 (C-FCR) to 0.62 (B-RD), all of which were not statistically significant (*p*s ranged from 0.034 to 0.195) and unacceptable (i.e., all ≤ 0.70). Sensitivities for these variables at previously validated cutoff scores ranged from 0.12 (B-RD ≤ 3) to 0.33 (C-FCR ≤ 15, C-RH ≤ 10, and C-RH ≤ 11). Specificity values generally hovered around 0.90, with the exception of C-FCR ≤ 15, which yielded lower specificity of 0.80. Classification accuracy ranged from 0.68 (C-FCR ≤ 15) to 0.77 (C-RH ≤ 10 and B-RH ≤ 4). Various cutoff scores and their sensitivity, specificity, and total accuracy values are displayed in Table 2.

As stated above, five arithmetic formulas were computed to determine if combining embedded PVT variables resulted in improved classification. AUCs for these variables ranged from 0.61 to 0.63 and were all nonsignificant compared to the conservative critical α (*p*s > 0.03) and unacceptable. Additionally, five logistic regressions (LRs) were performed with the combinations of variables described in the five formulas above entered as predictors as one set (i.e., all four were entered as predictors for the first, C-FCR and B-RH were entered as predictors for the second, etc.) with credible/noncredible group membership as the binary outcome variable. Exponentiated equations were derived from LR results, with similarly unacceptable AUC variables for each (0.61–0.64) such that were all nonsignificant compared to the conservative critical α (*p*s > 0.02). Due to these poor results and lack of potential clinical utility, cutoff scores and sensitivity/specificity values were not identified for these novel exponentiated equation variables as they appeared psychometrically inadequate.

## 4. Discussion

The present study sought to evaluate the clinical utility of four embedded performance validity test (PVT) variables in the CVLT-II and BVMT-R, commonly used in various clinical neuropsychological samples, in patients with MS. These variables were considered in isolation and in combination with both arithmetical and logistic regression-derived methods. Given the base rates of noncredible performance reported in patients with MS [18] and the important implications of non-credibility in neuropsychological assessment [25], the psychometric evaluation of PVTs in the neuropsychological assessment of patients in this population is necessary and clinically warranted [11]. Several findings deserve further discussion.

First and foremost, current findings extended prior work and indicated that general discriminability for several embedded CVLT-II and BVMT-R PVTs were unacceptably low, with none of the AUC values exceeding 0.70 (a generally accepted lower bound criterion denoting at least acceptable discriminability [43,50,51]) nor emerging as statistically significant compared to conservative, adjusted α. Additionally, a series of arithmetically- and logistic regression-derived formulas did not appear to bolster discriminability, despite recent work supporting these methods to derive useful multivariate composite formulas for this purpose [52]. In all, current findings suggested that these select embedded CVLT-II and BVMT-R PVTs are likely not appropriate in isolation nor in combination at detecting noncredible performance, let alone substitutable for standalone PVTs in this population.

Relatedly, current findings, at least in part, further replicate recent work on embedded PVTs in MS. Domen and colleagues [38] reported specificities for previously validated cutoff scores for C-FCR and B-RD ranged from 0.98–0.99 and 0.88–0.93, respectively. Current findings suggested specificities for these variables ranged from 0.80–0.90 and 0.90–0.92, respectively. Although the specificity for C-FCR ≤ 15 was 0.98 as reported by Domen and colleagues [38], these results suggested that 20% (i.e., specificity = 0.80) of the credible group were misidentified as noncredible, suggesting that a more conservative cutoff of ≤14 [37] may yield fewer false positives. Additionally, these results further extend Domen and colleagues’ [38] research as they revealed that specificities for C-RH and B-RH—neither of which were discussed by Domen and colleagues [38]—hovered around 0.90 (0.89–0.95). Current findings support their conclusion that various previously validated cutoff scores may avoid excessive false-positive errors in patients with MS. Nonetheless, despite broadly adequate levels of specificity for the embedded PVTs in isolation, their *sensitivities* were notably lacking, such that they likely do not have sufficient ability to detect the “signal” of noncredible performance during the neuropsychological assessment of pwMS. The embedded PVTs herein appeared to fall short of the “Larrabee limit” [28] (p. 1088), which states that sensitivities tend to hover around 0.50 while maintaining specificities of ≥0.90. Such psychometric considerations may result in false negatives in clinical decision-making (i.e., concluding that a patient is providing credible performance when he or she is not).

Importantly, current findings highlight a glaring insufficiency in recommended neuropsychological procedures and care regarding the evaluation of pwMS. Of note, neither the National MS Society’s recommendations for neuropsychology [1] nor the articles proposing the MACFIMS [9] and BICAMS [10] discuss the need for nor the role of performance validity assessment in MS. Only the MEMS [11] clearly identified and thoroughly discussed the continued need for this venture, though it did not explicitly recommend the inclusion of a standalone PVT in its battery. These findings indicate that embedded PVTs within the aforementioned batteries, which are certainly effective at reducing the time needed to complete neuropsychological testing, may be insensitive to noncredible performance.

In line with emerging literature on this topic [18,38], the authors strongly recommend that clinicians who perform neuropsychological evaluations with patients with MS include at least one standalone PVT in their battery and consider clinical guidelines in interpreting one or more data suggesting noncredible performance [53]. Performance on PVTs may account for extraordinary amounts of variance in neuropsychological test scores [54,55,56]. The reliance on invalid neuropsychological data may result in incorrect clinical conclusions with implications regarding patients’ continued medical care (e.g., receiving unwarranted medical treatment/diagnosis) [24,57,58] or extra-medical considerations (e.g., in the context of disability application or medicolegal assessments) [59,60].

From a clinical perspective, ideally, the choice of which PVT(s) to use will be made on the basis of the best available evidence and clinical judgment according to each patient’s presenting problems and neuropsychologist’s expertise. As an aside, the authors believe that the VSVT may be appropriate given its routine use in previous MS literature [18,21] and reported robustness against bona fide deficits in processing speed, working memory, and memory [42], each of which may be jeopardized in patients with MS.

### Limitations

The present study is not without limitations. First, noncredible performance was defined by the below criterion scores on a single, standalone PVT. Although the PVT chosen is widely used clinically and is psychometrically robust at detecting the “signal” of non-credibility without being negatively affected by bona fide weaknesses in aspects of processing speed, working memory, and attention [42], this methodological decision nonetheless may be critiqued in light of work highlighting multivariate models that rely on more than one datum to detect non-credibility [52,61]. Future work should seek to include enough psychometrically appropriate standalone and embedded PVTs to be consistent with these recommendations. Furthermore, these results focus exclusively on the CVLT-II and not the newly published CVLT-3 [62], which is broadly similar in nature albeit with differences most meaningfully in its forced-choice recognition items (which are not described in any detail herein for test security). Furthermore, the PVTs of interest (both standalone and embedded) utilized memory-based paradigms. It may be that PVTs tapping non-memory-based abilities (e.g., attention, visuospatial ability, language) provide improved classification statistics and deserve investigation. Additionally, given the multiplicity of variables that may play a role in noncredible presentations (e.g., psychiatric symptomatology, presence of secondary gain), future research is strongly encouraged to parse out the relevant dimensions that may meaningfully contribute to and/or explain noncredible performance on cognitive tests. Finally, while differences in gender and years of education between credible and noncredible groups were not considered to be clinically meaningful in this study (as is consistent with prior research with similar aims) [45,46,47], future work may seek to consider the impact of baseline patient characteristics on neuropsychological test scores and, by extension, performance validity in pwMS, as some have suggested that level of education and/or disease subtype may differentially influence aspects of test performance in MS [63].

## 5. Conclusions

In all, the present paper is the first to explore the possible clinical utility of four embedded PVTs in the CVLT-II and BVMT-R specifically within a sample of pwMS. Current findings revealed fairly poor classification statistics for these variables and highlight the need for a psychometrically sound assessment of performance validity in this population. The authors encourage clinicians who work with pwMS in a neuropsychological context to not rely solely on the available embedded metrics explored herein, but rather to routinely utilize standalone PVT(s) to maximize the interpretive quality of their cognitive data. Furthermore, the authors recommend that researchers explore the utility of various types of PVTs in pwMS.

## Figures and Tables

**Table 1:** Demographic characteristics of credible and noncredible groups.

	Credible (*n* = 100)	Noncredible (*n* = 33)	*χ*^2^ (*p*) or *U* (*p*)
	*M* (*SD*) or *n* (%)		
Age, Years	48.83 (13.13)	47.18 (10.67)	0.25 (0.76)
Gender	-	-	7.30 (**0.01**)
*Male*	29 (29)	2 (6)	-
*Female*	71 (71)	31 (94)	-
Education, Years	14.36 (2.49)	12.76 (2.11)	1020.00 (≤**0.01**)
Racial/Ethnic Identity	-	-	0.29 (0.59)
*White*	84 (84)	29 (88)	-
*Non-White*	16 (16)	4 (12)	-
MS Subtype	-	-	0.91 (0.64)
*Relapsing-Remitting*	75 (75)	25 (76)	-
*Secondary Progressive*	11 (11)	2 (6)	-
*Primary Progressive*	14 (14)	6 (18)	-
Symptom Duration, Years (*n* = 118)	14.32 (10.93)	10.18 (8.54)	979.50 (0.08)

*N* = 133 unless otherwise noted. *p* values displayed in boldface are considered significant at the adjusted criterion α of 0.013.

**Table 2:** Embedded CVLT-II and BVMT-R variables and classification statistics.

Variable	Raw Cutoff	AUC (*p* ^1^)	Credible *M* (*SD*)	Noncredible *M* (*SD*)	*U* (*p* ^1^), *d* ^2^	Sen.	Spec.	Acc.
C-FCR	≤15 ^a^	0.58 (0.195)	15.58 (1.03)	14.97 (1.98)	1401.50 (0.080), 0.22	0.33	0.80	0.68
	≤14 ^b^	-	-	-	-	0.24	0.90	0.74
C-RH	≤11 ^c^	0.61 (0.066)	13.71 (2.36)	12.45 (3.16)	1296.50 (0.062), 0.32	0.33	0.89	0.75
	≤10 ^c^	-	-	-	-	0.33	0.91	0.77
B-RH	≤4 ^d^	0.60 (0.096)	5.66 (1.28)	5.15 (1.28)	1330.50 (0.042), 0.29	0.24	0.95	0.77
B-RD	≤4 ^e^	0.62 (0.034)	5.44 (0.88)	4.85 (1.46)	1244.00 (0.017), 0.37	0.30	0.90	0.75
	≤3 ^d^	-	-	-	-	0.12	0.92	0.72

Credible *n* = 100. Noncredible *n* = 33. ^1^ Uncorrected p value reported, with conservative critical α of 0.013 chosen to interpret statistical significance to minimize Type 1 error. ^2^ Cohen’s d effect size converted from Mann-Whitney U values. [48]. ^a^ [49]. ^b^ [37]. ^c^ [34]. ^d^ [35]. ^e^ [33]. C-FCR = California Verbal Learning Test, Second Edition (CVLT-II) Forced Choice Recognition. C-RH = CVLT-II Recognition Hits. B-RH = Brief Visuospatial Memory Test, Revised (BVMT-R) Recognition Hits. B-RD = BVMT-R Recognition Discrimination. Sen. = Sensitivity. Spec. = Specificity. Acc. = Overall classification accuracy.

## Data Availability

The data presented in this study may be available on reasonable, approved request from the corresponding author.
